# Differences between Spatial and Visual Mental Representations

**DOI:** 10.3389/fpsyg.2013.00240

**Published:** 2013-05-08

**Authors:** Jan Frederik Sima, Holger Schultheis, Thomas Barkowsky

**Affiliations:** ^1^SFB/TR 8 Spatial Cognition, Universität BremenBremen, Germany

**Keywords:** mental representation, mental imagery, mental models, preferred mental models, visual mental representation, spatial mental representation, eye tracking

## Abstract

This article investigates the relationship between visual mental representations and spatial mental representations in human visuo-spatial processing. By comparing two common theories of visuo-spatial processing – mental model theory and the theory of mental imagery – we identified two open questions: (1) which representations are modality-specific, and (2) what is the role of the two representations in reasoning. Two experiments examining eye movements and preferences for under-specified problems were conducted to investigate these questions. We found that significant spontaneous eye movements along the processed spatial relations occurred only when a visual mental representation is employed, but not with a spatial mental representation. Furthermore, the preferences for the answers of the under-specified problems differed between the two mental representations. The results challenge assumptions made by mental model theory and the theory of mental imagery.

## 1 Introduction

Our everyday behavior relies on our ability to process visual and spatial information. Describing the route to work, taking another person’s perspective, or imagining a familiar face or object all depend on our capability to process and reason with visual and spatial information.

Two main theoretic frameworks of visual and spatial knowledge processing have been proposed in cognitive science: mental model theory (Johnson-Laird, 1989, 1998; Tversky, 1993) and mental imagery (Finke, 1989; Kosslyn, 1994; Kosslyn et al., 2006). Furthermore, there is also the conception of verbal or propositional mental representations (Rips, 1994; Pylyshyn, 2002) that employ a sort of logical inference to reason about visual and/or spatial information. However, considerable evidence indicates that analogical mental representations, i.e., mental models or mental images, can better predict and explain the empirical data, in particular, for spatial reasoning (e.g., Byrne and Johnson-Laird, 1989; Kosslyn, 1994; Johnson-Laird, 2001).

In line with behavioral and neuroscientific evidence (e.g., Ungerleider and Mishkin, 1982; Levine et al., 1985; Newcombe et al., 1987; Farah et al., 1988; Courtney et al., 1996; Smith and Jonides, 1997; Mellet et al., 2000; Knauff and Johnson-Laird, 2002; Klauer and Zhao, 2004), mental model theory and the theory of mental imagery both propose a distinction between spatial and visual mental representations. The theory of mental imagery proposes spatial mental images and visual mental images; mental model theory proposes (spatial) mental models and visual mental images. Research based on the theories has, however, mostly focused on one of the two representations: the investigation of the properties of visual mental images in the theory of mental imagery and the investigation of reasoning with (spatial) mental models in mental model theory. Consequently, the relationship and interaction between the two types of mental representations is left largely unspecified in both theories. Although initial attempts exist (e.g., Schultheis and Barkowsky, 2011) to explain how visual and spatial mental representations interact and relate to each other, empirical data on the issue is largely missing. Accordingly, the primary aim of this article is to examine the differences and the relationship between visual and spatial mental representations. To achieve this, we first review how mental model theory on the one hand and the theory of mental imagery on the other hand understand spatial and visual mental representations as well as how they interpret the relationship between them. Even though there is much theoretic and empirical work on both theories, the literature lacks a systematic comparison of the theories. In the following, we present such a comparison. From this comparison, it will become clear that the theories actually propose very similar conceptions of spatial and visual mental representations but that their foci of investigation are mostly on different aspects and include phenomena not investigated within the respectively other theory. We examined these different aspects and used them in our experiments to gain new insights into the open issues of the relationship between visual and spatial mental representations. The results can be applied to complement gaps in the two theories.

## 2 Theories

### 2.1 Mental model theory

Mental model theory (Johnson-Laird, 1998) postulates that there are three representational levels involved in human thinking: propositional representations, mental models, and mental images. The relationships between these three levels are hierarchical in the sense that their construction depends on each other. The following example helps to illustrate this point. Three-term series problems (Johnson-Laird, 1972) are common experimental tasks in the study of mental models. They contain two premises and one conclusion that has to be validated or inferred based on the premises. Let the two premises be “A is left of B” and “B is right of C” and let the to-be-drawn conclusion be the relationship between A and C. According to mental model theory the premises are first encoded propositionally. From these propositional premises a mental model of the described configuration is constructed. As it is an essential property of mental models that “the structural relations between the parts of the model are analogous to the structural relations in the world” (Johnson-Laird, 1998, p. 447), one valid mental model of our example can be depicted in the following way:

A        C        B

We note that a mental model is a special case of the situation defined by the premises, because it only represents one valid situation with respect to the premises. For our example another mental model that satisfies the premises is:

C        A        B

Just like the situation represented by a mental model is a special case of what is described in the premises, mental model theory poses that a mental image is a special case of a given mental model. The mental image that is constructed from a mental model is one specific instance out of many valid instances described by the model, because the image has to specify, for example, the distance between the entities. The underlying mental model is in contrast invariant with respect to the distances. Summarizing the hierarchical structure of mental model theory, we note that a mental image is one out of many projections of the visualizable aspects of a mental model, and a mental model is one out of many analogically structured configurations that are valid given the propositionally represented premises. This suggests a clear hierarchy in which it is necessary to have the more general representations in order to construct a more specific one.

Mental models are described to be analogically structured, amodal, and abstract, e.g., they can represent abstract, non-visualizable relations such as “smarter than.” In contrast, mental images can only represent “visualizable” information and are modality-specific to visual perception (e.g., Johnson-Laird, 1998; Knauff and Johnson-Laird, 2002). It has been suggested that the analogical nature of mental models might be generally spatial (Knauff et al., 2003), i.e., even reasoning with abstract relations like “worse than” or “better than” is handled by a spatio-analogical mental model. This view is supported by the association of mental model reasoning with activation in the parietal lobe (e.g., Goel and Dolan, 2001; Knauff et al., 2003), which is associated with several processes of spatial cognition (for an overview, see Sack, 2009). It was found that the use of “visual” relations, e.g., “dirtier than,” in relational reasoning tasks led to activation in the early visual cortex in contrast to tasks with other (abstract) relations, e.g., “worse than” (Knauff et al., 2003). The study also found that “visual” relations led to longer reaction times and it was concluded that tasks using such “visual” relations induce the employment of visual mental images during the mental-model-based reasoning process.

Most of the literature on mental model theory focuses on how mental models explain reasoning. Johnson-Laird and Byrne (1991) state that reasoning according to the mental model theory consists of three stages: (1) the construction of one mental model (construction phase), (2) the inspection of the mental model (inspection phase), and (3) the variation of the mental model (variation phase). Slightly simplified, the reasoning process works as follows. One first mental model is constructed based on the given premises. This model represents one situation that is valid given the premises. This situation is inspected and can yield a possible conclusion. This conclusion is then verified to be valid in all other possible mental models that can be derived from the premises. If a conclusion is not contradicted in the other valid mental models, the conclusion is confirmed. There is much empirical support for this three stage process in human reasoning (e.g., Johnson-Laird, 2001). One interesting phenomenon in reasoning with mental models is the occurrence of preferred mental models when there are multiple valid conclusions. An example for such multiple valid conclusions are the two configurations “CAB” and “ACB” of the above example. It can be observed that there are reliable within-subject and between-subject preferences for which model is constructed first out of several valid mental models. This firstly constructed mental model is termed a preferred mental model. As a consequence, if there are several valid conclusions that can be inferred, there is a preference for one conclusion which corresponds to the preferred mental model. Preferred mental models have been investigated in different domains, but in particular in the domain of spatial reasoning (e.g., Rauh et al., 2005; Jahn et al., 2007; Schultheis and Barkowsky, 2013).

### 2.2 Theory of mental imagery

The theory of mental imagery (Kosslyn, 1994; Kosslyn et al., 2006) makes a distinction between spatial mental images and visual mental images. These two mental representations differ in the content they represent and are distinct in their anatomical localization. But they are both assumed to have a (at least partially) spatio-analogical structure. Furthermore, there is also a propositional representation referred to as associative memory, which contains propositional descriptions of the structure of an object or a scene. This information can be used to construct spatial and visual mental images. For the latter, however, one needs to further retrieve encoded shape information from another source, i.e., the object-properties-processing subsystem, which can be thought of as a sort of non-analogical visual memory store located in the temporal lobe.

Spatial mental images (sometimes referred to as object maps) are located in the spatial-properties-processing subsystem in the framework of Kosslyn (1994). They contain information about the location, size, and orientation of entities. The spatial-properties-processing subsystem is (at least partially) placed in the parietal lobe. Given that areas of the parietal lobe are topographically organized (Sereno et al., 2001), it is assumed that spatial mental images are also at least partially spatio-analogical (Kosslyn et al., 2006).

Visual mental images are constructed and processed in a structure called the visual buffer. The visual buffer consists of the topographically organized areas of the visual cortex. Visual mental images are thus assumed to be spatio-analogical or “depictive,” i.e., the metrics of what is represented, e.g., a shape, are reflected in the metrics of the representation. Visual mental images represent shape information, as well as, for example, color and depth.

A difference between spatial and visual mental images is that spatial mental images contain more information, in the sense that the current visual mental image in the visual buffer only contains a “visualized” part of what is represented in the spatial mental image (Kosslyn et al., 2006, p. 138). A visual mental image is a specification of a part of a spatial mental image.

Four types of functions are proposed for visual and spatial mental images: generation, inspection, maintenance, and manipulation. The generation of a mental image can either be just the retrieval of a spatial configuration of entities as a spatial mental image if no visual information is necessary for a given task or it can furthermore include the retrieval of shape information to generate a visual mental image in the visual buffer. Note that the visual buffer does not need to be employed for spatial mental images. Kosslyn et al. (2006) states that the processing of spatial and visual mental images occurs in parallel, i.e., the image of a shape is generated while a spatial image is generated. They furthermore state that this parallel processing might not always be useful, as the proper construction of a shape requires information about its spatial properties, i.e., location, size, and orientation which are provided by a respective spatial mental image (Kosslyn et al., 2006, p. 143). For the generation of multi-part visual mental images, a corresponding spatial mental image is necessary to guide the placement of shapes in the visual buffer by specifying the location, orientation, and size. The inspection process can make previously implicit information in a visual or spatial image explicit, i.e., new information is inferred. Visual mental images are inspected by shifting an attention window over the visual buffer. Through this inspection visual information, e.g., properties of a shape, as well as spatial information, e.g., spatial relations, can be inferred. It is also possible that new information is inferred from only a spatial mental image. However, no detailed information on the inspection of/inference in spatial mental images is provided by the theory. The function of image maintenance is used to re-construct parts of mental images as the information fades over time. The function of image manipulation allows the imagination of transformations of mental images. The theory posits that such manipulations are realized by altering the object map, i.e., the spatial mental image, underlying the visual mental image. One would, for example, change the location or size of an entity in the spatial mental image to alter the visual mental image that contains the shape information of that entity.

One interesting phenomenon of mental imagery is the observation of spontaneous eye movements during different visual mental imagery tasks. Brandt and Stark (1997) had participants imagine a previously memorized grid pattern and found that the eye movements during imagination reflected the content of the original stimuli. Spontaneous eye movements that reflect the processed spatial relations during mental imagery have since been found, for example, during imagination of natural scenes (Holsanova et al., 1998), during imagination of detailed paintings and detailed descriptions of scenes while facing a white board as well as in total darkness (Johansson et al., 2006), during reasoning with “visual” syllogisms, e.g., “a jar of pickles is below a box of tea bags,” (Demarais and Cohen, 1998), and while listening to verbal descriptions of spatial scenes, e.g., “at the bottom there is a doorman in blue” (Spivey and Geng, 2001). Johansson et al. (2012) report a series of experiments, in which participants were selectively forced to not move their eyes during mental imagery. They found that the suppression of eye movements has an impact on the quantity and quality of mental imagery. Their results strongly indicate a functional role of eye movements during mental imagery.

### 2.3 Open questions

The previous two sections are summarized in Table 1 which provides a comparative overview of the two theories. From the comparison of the two theories, a great overlap in the assumptions made and structures and processes proposed by the two theories is evident. Many aspects of the two theories are revealed to be rather similar, perhaps more similar than one would have expected. In particular, they provide very similar descriptions of a spatial and a visual mental representation with respect to information content, localization, and hierarchical structure between the two representations. There are, however, some diverging predictions with respect to the modality of these representations and their role in reasoning. In the following, we discuss these differences and identify two main questions that arise from the comparison of these two theories.

**Table 1:** **Comparison of mental model theory and the theory of mental images**.

	Mental model theory		Mental imagery	
	Mental model	Mental image	Spatial mental image	Visual mental image
Structure	Structurally analogical to problem domain (Johnson-Laird, 1998); amodal or multi-modal (Knauff and Johnson-Laird, 2002); spatio-analogical (Knauff et al., 2003)	No concrete statements about structure are made	Spatio-analogical (Kosslyn et al., 2006); described as configuration of points in space (Kosslyn, 1994, p. 324)	Spatio-analogical, i.e., “depictive” (Kosslyn, 1994)
Anatomical localization	Parietal lobe plays a key role in mental model reasoning (Knauff et al., 2003)	Occipital lobe (specifically V2) (Knauff et al., 2003)	Posterior parietal lobe (Kosslyn et al., 2006)	Topographically organized areas of the occipital lobe (the visual buffer) (Kosslyn and Thompson, 2003)
Relationship between the two representations	Mental images are special cases of mental models (Johnson-Laird, 1998). Reasoning is realized with mental models (Knauff and Johnson-Laird, 2002)		Spatial mental images (object maps) set spatial parameters, e.g., location, size, and orientation for the shapes represented in a visual mental image (Kosslyn et al., 2006); a visual mental image represents a “visualized” part of a spatial mental image (Kosslyn et al., 2006, p. 138)	
Content	Abstract relations, e.g., ownership, “worse than,” and spatial relations, e.g., orientation, distance, topology (for an overview, see Johnson-Laird, 2001)	Visual information, e.g., visual configuration seen from a certain perspective (Johnson-Laird, 1998)	Spatial properties, e.g., location, size, orientation (Kosslyn et al., 2006)	Visual/object properties, e.g., shape information, color, depth (Kosslyn et al., 2006)
Processes	Model construction, model inspection, model variation (Johnson-Laird and Byrne, 1991)	Mental images can be constructed from visualizable parts of an underlying mental model (Johnson-Laird, 1998); insights from image manipulation are reinterpreted within the underlying mental model (Johnson-Laird, 1998)	Construction, inspection, maintenance, manipulation (Kosslyn, 1994); inspection (including inference of new information) of visual mental images is explained by employing processes of visual perception on the content of the visual buffer; inference from spatial mental images is possible	
Typical experimental paradigms	Different (often spatial) syllogisms without any references to visual imagination (for an overview, see Johnson-Laird, 2001)	Syllogisms with visual but non-spatial relations, e.g., “dirtier than” (Knauff and Johnson-Laird, 2002)	To our knowledge there is no paradigm to specifically induce spatial mental images	“Imagine,” “try to see mentally” (e.g., Kosslyn, 1973, 1980; Chambers and Reisberg, 1985; Borst et al., 2006)
Phenomena unique to theory	Preferred mental models (e.g., Jahn et al., 2007)		Spontaneous eye movements corresponding to the processed content in mental images (e.g., Johansson et al., 2006, 2012)	

The theory of mental imagery states that spatial mental images are processed in a component called the spatial-properties-processing subsystem. This subsystem is explicitly linked to the dorsal processing stream, which processes spatial information during visual perception (Kosslyn et al., 2006, p.138). Processing of spatial mental images uses (at least partly) the same processes used during processing of spatial information in visual perception. Mental models on the other hand are commonly assumed to be amodal or multi-modal (e.g., Johnson-Laird and Byrne, 1991). Accordingly, mental models are assumed to be used to also reason about abstract, non-spatial, information, e.g., “A is better than B” (Knauff et al., 2003), whereas spatial mental images are assumed to process only spatial information. It has, however, been assumed that abstract information, e.g., “better than,” can be translated into spatial information in mental models (Knauff et al., 2003). An opinion seemingly shared by Kosslyn (1994), who states that information like “A is smarter than B” can be represented by dots on a line in a spatial mental image which would then correspond to a mental model in the sense of Johnson-Laird (Kosslyn, 1994, p. 324). The question that remains is whether the spatial representation, described as a mental model or a spatial mental image, is actually amodal/multi-modal (as claimed by mental model theory) or linked to the modality of visual perception (as seemingly proposed by the theory of mental imagery). Results pointing either way would help refining the theories.

Another open issue is the theories’ seemingly different prediction on the role of the spatial mental representation in reasoning. Unfortunately, both theories remain vague regarding the details of how spatial and visual representations interact during reasoning. In mental model theory it is often explicitly stated that it is mental models and not mental images that underlie human reasoning (Knauff and Johnson-Laird, 2002; Knauff et al., 2003). The automatic generation of mental images through “visual” relations, e.g., “the dog is dirtier than the cat” is even considered to impede the reasoning process that happens on the level of mental models (Knauff and Johnson-Laird, 2002). Of course, mental images can be important for reasoning if certain visual information is necessary, but it is not described how such visual information would be interpreted by nor how it would be transferred into the mental model for further reasoning. In the theory of mental imagery, it is made clear that visual mental images play a major role in reasoning: “[I]magery plays a critical role in many types of reasoning” (Kosslyn, 1994, p.404). And, contrasting mental model theory, visual mental images are assumed to be much more than just the provider of visual information for spatial mental images, in general, and particularly in reasoning (Kosslyn, 1994; Kosslyn et al., 2006). The inspection of visual mental images constructed in the visual buffer can lead to new insights and is thus directly involved in the reasoning processes. According to Kosslyn et al. (2006) a visual mental image is generated using an underlying spatial mental image. However, the concrete role of the spatial mental image in the reasoning process is never elaborated in a way that would suggest that the spatial mental image is of specific importance to reasoning or even that it might be the actual reasoning component (as proposed in mental model theory).

Summarizing, we pointed out two main open issues regarding the differences between spatial mental representations and visual mental representations: (1) whether the spatial mental representation is rather amodal/multi-modal or whether it is also directly linked to visual perception like the visual mental representation; (2) to which extent the two mental representations are involved in reasoning, i.e., whether the spatial mental representation is the primary reasoning component or not.

## 3 Experiments

The comparison of the two theories, furthermore, showed that there are phenomena which have mostly been investigated only within the framework of one of the two theories. Preferences in under-specified problems have so far only been investigated within the framework of mental model theory while eye movements have been a focus of investigation almost only with mental images. In the presented experiments, we investigated to which extent these two phenomena are transferable to the respectively other type of mental representation. That is, we checked for spontaneous eye movements during reasoning with a spatial mental representation, i.e., a (spatial) mental model, and we checked for possible preferences when employing a visual mental representation, i.e., a visual mental image. In the following, we describe how the investigation of these phenomena informs us about the open questions stated in Section 3.3.

The tasks used in the experiments are three-term series relational reasoning problems about orientation knowledge. The two experiments differed only in their instructions which were formulated so that they induced the employment of a spatial mental representation in the first experiments and a visual mental representation in the second experiment.

We assume that we will confirm the findings of the literature that systematic eye movements occur during the second experiment (employing a visual mental representation) and that there are significant preferences in the answers of the participants in the first experiment (employing a spatial mental representation). The apparent functional role of eye movements during visual mental imagery provides strong evidence that visual mental representations are linked to processes of visual perception. These spontaneous eye movements reflect the spatial relations of the processed information. Both mental model theory and the theory of mental imagery assume spatial relations to be represented by a spatial mental representation, which supports the construction of a visual mental representation by providing the required spatial information. We tested whether such eye movements along the processed spatial relations would occur during employment of only a spatial mental representation, i.e., without the representation of visual content. The investigation of eye movements in this context can inform us about the question of the modality of spatial mental representations: if systematic eye movements occur during reasoning with spatial mental representations, then this would be a strong indication that mental models are not amodal, but are, in fact, linked to attentional processes of visual perception. A lack of systematic eye movements during reasoning with spatial mental representations, on the other hand, would support the assumption of mental model theory that mental models are amodal. More specifically, this would indicate that the processes of spatial mental representations do not employ the overt attentional processes of visual perception as it is the case for visual mental representations.

Preferred mental models are preferences for certain answers to under-specified reasoning problems that have been found for reasoning with mental models. These preferences are assumed to emerge because participants first construct one, perhaps the most parsimonious, mental model out of several valid models (e.g., Rauh et al., 2005). Visual mental images are also assumed to “depict” just one situation at a time; in fact it is hard to imagine how a “depictive” representation could represent more than one situation simultaneously. There are three possible outcomes for our investigation of such preferences for reasoning with visual mental representations: (1) we find no significant preferred answers, (2) we find different preferences for the two mental representations, or (3) we find the same preferences in reasoning with both mental representations. Finding no significant preferences in the answers when a visual mental representation is employed would strongly indicate that the assumption that visual mental representations build upon corresponding spatial mental representations is incorrect. Furthermore, this would indicate that the construction of visual mental representations can be subject to very strong individual differences. Such a finding seems unlikely and would not be predicted by any of the two theories. Should we find the same preferences in both experiments, i.e., for reasoning with both a spatial and a visual mental representation, the assumption of a hierarchal relationship between the two mental representations would be supported. This would strongly suggest that indeed the spatial configuration of a visual mental representation is taken from an underlying spatial mental representation. Should we find different preferences for the two mental representations, refinements of both mental model theory and the theory of mental imagery would be required to explain this disparity. In particular, such a finding would challenge the two theories to elaborate on their assumption that the construction of visual mental representations depends on an underlying spatial mental representation. Additionally, the strong claim made by mental model theory that reasoning is realized by spatial mental representations and not visual mental representations would without additional hypotheses be contradicted by this result.

In the following, the materials and methods employed in both conducted experiments are described.

### 3.1 Materials and apparatus

The tasks used in the experiments are under-specified three-term series problems about orientation knowledge, specifically cardinal directions. We chose these problems because problems of this type, i.e., three-term series relational spatial reasoning, have been used in several studies of mental model theory before (e.g., Knauff et al., 2003; Byrne and Johnson-Laird, 1989; Schultheis et al., in revision). We use an eight-sector model of cardinal directions, i.e., the eight directions are north, north-east, east, south-east, south, south-west, west, and north-west. The problems are of the following form:

Premise 1: A is [direction 1] of B, e.g., *A is north of B*
Premise 2: B is [direction 2] of C, e.g., *B is east of C*
Conclusion: As seen from A, where is C?

**Figure 1:**
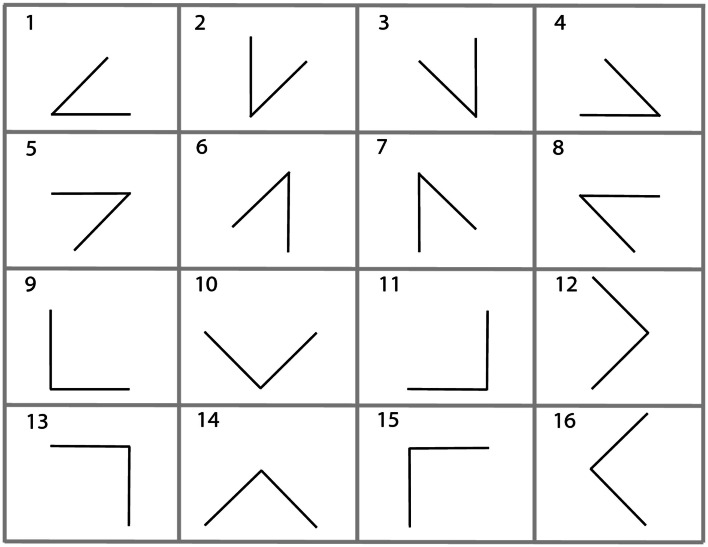
**The 16 different types of problems used in the experiments**. The upper eight are 45° problems and the lower eight are 90° problems.

The premises provide two spatial relations between three entities and the third spatial relation has to be inferred. In general, these problems are under-specified, i.e., there can be more than one correct conclusion given the premises. We used two classes of these problems, which we term 45° problems and 90° problems. These problems can be visualized as triangles with one of the three edges missing. This missing edge corresponds to the to-be-inferred spatial relation. We used all possible combinations in which the two given edges form either a 45° or a 90° angle. Figure 1 depicts an overview of all these problems.

**Figure 2:**
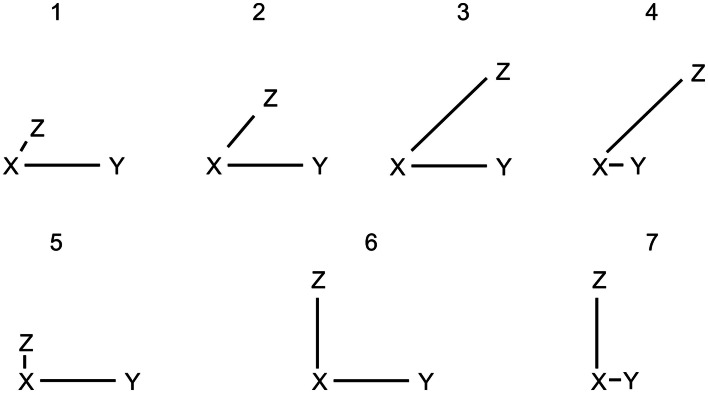
**Possible valid models for a 45° problem are depicted as 1, 2, 3, and 4**. Possible valid models for a 90° problem are depicted as 5, 6, and 7. The models 1, 4, 5, and 7 are termed distorted models (DM) because the distances between the entities vary a lot from each other. The models 2 and 6 have equal distances and are termed equal-distance models (EDM). The model 3 is termed cardinal model (CM) because the to-be-inferred relation corresponds to one of the main cardinal directions, i.e., north, east, south, or west.

We can identify all possible correct solutions for the two problem sets. The 90° problems have three possible solutions and the 45° problems have 4 possible solutions. The different configurations leading to these solutions are depicted in Figure 2. To distinguish the different solutions, we classify the underlying mental representations based on a visualization of the solution as triangles. In this context we use the term “model” to describe the underlying mental configuration, whether it might be a spatial mental representation or a visual mental representation. Models with very different distances for the given spatial relations are termed *distorted models* *(DM)*; models with roughly equal distances for the given relations are termed *equal-distance models (EDM)*. The remaining valid solution for the 45° problems, the third solution in Figure 2, always leads to one of the four main cardinal directions being inferred and is therefore termed *cardinal model* *(CM)*.

There are 16 different possible problems. We used them all twice with different letters resulting in a total of 32 problems. The 16 different problems consist of eight 45° and eight 90° problems, as depicted in Figure 1.

Participants wore a head-mounted SensoMotoric Instruments (SMI) iView X HED eye tracker with a 200 Hz sampling rate to record their eye movements. To prevent expectancy effects, participants were told that the experiment investigates the size of their pupils. A post-experimental questionnaire verified that participants were not aware of the eye tracking.

### 3.2 Procedure

#### 3.2.1 Instructions

The two experiments used slightly different instructions, so that they conformed with the usual instructions of both studies on mental models as well as studies on visual mental images. At the same time, the minimal change between the experiments helped to keep the tasks as similar as possible and minimize any differences besides the induced mental representation.

The instructions of the first experiment did not contain any suggestions to use visualization or visual information, but simply asked participants to infer the missing relation as fast and as accurately as possible. It is in line with previous experimental studies to assume the employment of mental models, i.e., a spatial mental representation, based on the fact that no visual information is required, given or asked for in the task (e.g., Johnson-Laird, 2001; Knauff and Johnson-Laird, 2002; Jahn et al., 2007).

The instructions of the second experiment only differed slightly from those of the first one. The participants were told that the letters represent cities that are to be imagined as little red squares with the respective letter next to them, which are all placed on a map. This slight variation made the instructions conform with those of several other visual mental imagery studies, i.e., using phrases such as “imagine […]” or “try to mentally see […]” (e.g., Kosslyn et al., 1983; Chambers and Reisberg, 1985; Borst et al., 2006).

In both experiments participants were asked to work as accurately and as fast as possible.

#### 3.2.2 Setup

Participants were seated facing a blank white wall at a distance of approximately 1 m. Their hands were placed on their legs under a table holding a computer mouse in the one hand and a small ball in the other one. This was to prevent participants from using their fingers as an aid to solve the tasks. The eye tracker was mounted on the participant’s head and calibrated. All initial instructions of the experiment were projected on the white wall.

#### 3.2.3 Learning phase

The experiment started with a learning phase to familiarize the participants with the cardinal directions. The learning phase consisted of acoustically presented statements and an answer screen with a question. Each statement was of the form “*K is [direction] of U*.” After 4 s the answer screen appeared, which depicted the reference entity U surrounded by the numbers 1 to 8 in a counterclockwise circular order together with the question “*As seen from U, where is K?*” The eight numbers represented the eight cardinal directions (1 = north, 2 = north-west, 3 = west, … 8 = north-east). Participants answered by naming the respective number. In case of an incorrect answer, the correct answer was projected on the wall. The training phase ended as soon as each of the eight cardinal directions was recognized correctly twice in a row.

#### 3.2.4 Problem trials

Participants were presented with a total of 48 trials. Out of those the first four were pre-trials intended to familiarize the participants with the form and procedure of the problems. Out of the remaining 44 trials, 12 were designed as filler trials. These filler trials differed in the order in which the entities were presented: AB, AC, BC, e.g., “*A is north of B; A is west of C; B is? of C*,” in contrast to the order of the remaining 32 problem trials: AB, BC, CA, e.g., “*A is north of B; B is east of C; C is? of A*.” The filler trials served a double purpose. First, they were meant to prevent memory effects due to the identical order of all problem trials. Second, filler trials were employed to identify those time intervals in which participants show eye movements along the given directions. We elaborate on this method in Section 4.3. After the presentation of the four pre-trials, the remaining 44 trials were presented in randomized order.

#### 3.2.5 Presentation

All premises and questions were presented acoustically. There was no projection on the white wall during the premises; after the conclusion phase an answer screen was projected onto the wall. Participants used the mouse to trigger the acoustic presentation of the first premise in each trial. As soon as they understood the statement, they clicked again for the presentation of the second premise. Similarly, they triggered the acoustic presentation of the question after having understood the second premise. Only after participants found an answer, they clicked the mouse again making the answer screen appear. The answer screen was the same as the one used in the learning phase. Participants verbally gave their answer by naming the number associated with the resulting direction. Participants continued to the next trial by clicking the mouse again.

The participants took between 35 and 50 min to complete the experiment.

### 3.3 Processing of the eye tracking data

We processed the eye tracking data to identify whether eye movements occurred along the spatial relations given in each trial. We employed the same method for both experiments.

The raw eye tracking data collected by the iView X software was first converted using the IDF Event Detector to generate a list of fixations made by the participant. Saccades were calculated automatically from the sequence and coordinates of the participant’s fixations. Using the starting and ending coordinates of each saccade, we classified them into one of eight categories corresponding to the eight cardinal directions used in the trials. All possible angles of a saccade, interpreted as a vector in a Cartesian plane, were uniformly mapped to the set of cardinal directions. Each direction corresponds to a range of angles on the degree circle with each direction taking up (360°/8) = 45°. For example, *north* corresponded to all angles in the range of 0° ± (45°/2) = 0° ± 22.5° = [337.5°; 22.5°]. Note that the eye movements classified in this way are *relative* eye movements, i.e., the absolute coordinates do not matter. This is reasonable considering that participants moved their head during trials and that arbitrary eye movements occurred in between. Given this classification, we were able to investigate a possible coupling between the given direction and observed eye movements during a trial. If eye movements are linked to the processing of spatial relations, we expected eye movements to occur not only along the given direction, but also along the opposite one. Assuming a mental representation of, for example, A being north of B, it is plausible to not only expect attention shifts from A to B but also from B to A during inspection as well as construction of the representation. Thus, we always compared the absolute number of observed saccades to the sum of saccades made along the given and the opposite direction. For the first premise, we used the given direction, e.g., for the premise *A is north of B* we looked for saccades along the north-south axis. For the second premise, we used the direction given in the first premise as well as the new direction given in the second premise, e.g., for *B is west of C*, we looked for north-south (from premise 1) and for east-west. For the conclusion phase, we used the direction (and its opposite) that was given as the answer by the participant. We applied a binomial test with a probability of 1/4 to test whether the two expected directions were above chance for each participant for the first premise and the conclusion. For the second premise we applied a binomial test with a probability of 1/2 to test whether the four expected directions (two directions from each relation of the two premises) were above chance. For each phase we then applied a binomial test with a probability of 0.05 to check whether the number of participants showing significant eye movements is significantly above chance. The probability of 0.05 corresponds to how often a false positive of the previous binomial test is expected.

No prior information was available on when during the processing of the premises or the conclusion eye movements are to be expected. It is likely that participants spent some time understanding and verbally processing the presented premise or question before they started constructing the mental representation. Similarly, participants required some time preparing the action of clicking the mouse to trigger the next step after they finished the processing of the respective premise or question. We, therefore, used the obtained data during the first premises of the filler trials to gather information on when exactly participants started showing eye movements and whether we could find a temporal pattern. We only looked at eye movements during the first premise, because the filler trials are identical to the problem trials for the first premise. The difference in the order of the presented letters only became evident with the second premise. Therefore, we assumed the same behavior in the first premises of both the problem and the filler trials. We looked at the time interval between the first mention of the direction in the first premise and the time participants click to initiate the second premise. This interval was divided into ten equally long time slots. For each of these ten slots we summed up the eye movements of all participants for each experiment. We checked whether eye movements along the expected directions, i.e., those given in the respective premise (and its opposite), were significantly above chance in each of these intervals. We applied a binomial test using a probability of 1/4 for each of the four pairs of cardinal directions, e.g., north/south compared to east/west, north-east/south-west, and north-west/south-east. We applied this method independently for both experiments and used the identified time slots for the eye movement analysis of the problem trials.

### 3.4 Ethics statement

The study was conducted within the Collaborative Research Center Spatial Cognition SFB/TR 8 funded by the German Research Foundation (DFG). The DFG’s board of ethics passed the research proposal that underlies the present study. DFG-funded projects do not require additional approval by other ethics committees. The studies are in full agreement with the ethical guidelines of the German Psychological Society (DGPs). Written informed consent was acquired from all participants.

## 4 Results

### 4.1 Experiment 1: Spatial mental representation

#### 4.1.1 Participants

Thirty undergraduate students of the University of Bremen, 12 male and 18 female, volunteered to take part in the experiment for monetary compensation.

Out of the 30 participants, one aborted the experiment and four were discarded due to an error rate of more than 30% incorrectly answered trials. The remaining 25 participants comprised 11 males and 14 females. The 0.05 level of significance was used for all statistical tests in both experiments.

#### 4.1.2 Preferences

**Figure 3:**
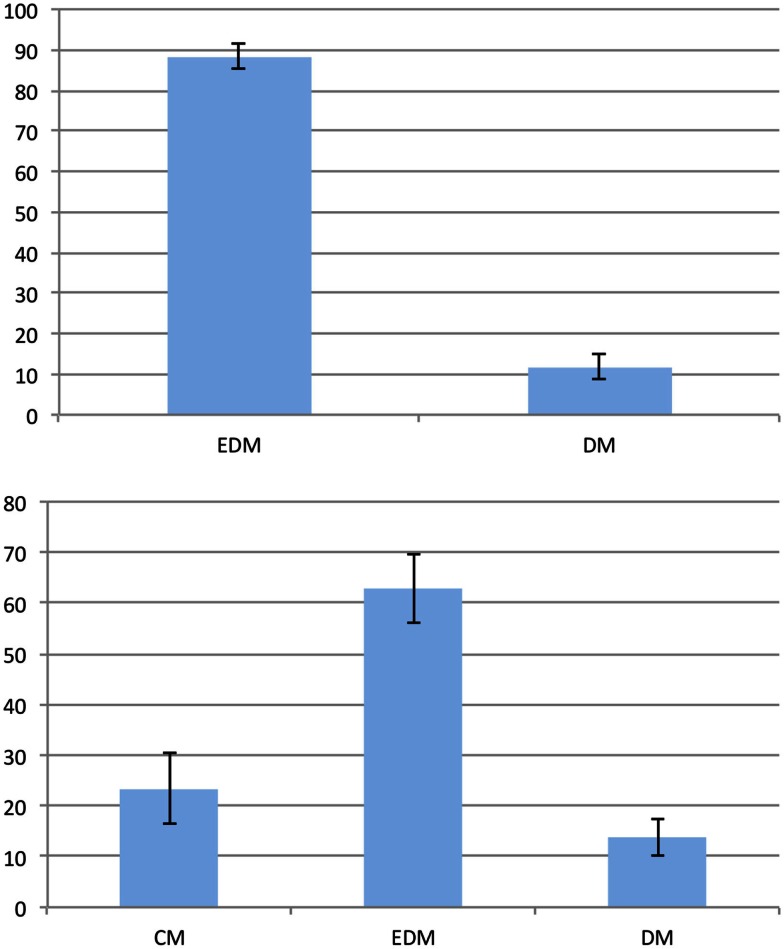
**Preferences in the first experiment**. The vertical axis represents the frequency of the given answer. Top: 90° problems; bottom: 45° problems. Error bars show the standard error of the mean. EDM, equal-distance model; CM, cardinal model; DM, distorted models.

For the analysis of the preferences we discarded those trials for which the participants gave no or incorrect answers (12% of all trials). We compared the answers of all participants for all remaining trials to identify possible preferences. We differentiated between 90° and 45° problems and assumed that the given answers indicate the employment of the corresponding model. If no preferences existed, one would expect to observe distorted models and equal-distance models in 66% and 33% of all 90° problem trials, respectively. Likewise, distorted models, equal-distance models, and cardinal models should occur in 50%, 25%, and 25% of all 45° problem trials, respectively. To check for the existence of preferences, we compared the observed model percentages to these hypothetical ones. Figure 3 shows the resulting preferences for both problem types. There is a clear preference for the equal-distance model in the 90° problems. The answer corresponding to this model was given in 88.34% of all trials (*t*_(24)_ = 17.233; *p* < 0.001). The distorted models were employed significantly less than expected by chance with 11.66% (*t*_(24)_ = −17.233; *p* < 0.001). We found a significant preference for the equal-distance model in the 45° problems with 62.88% (*t*_(24)_ = 5.352; *p* < 0.001), whereas the 23.46% of the cardinal model did not differ significantly from the expected value (*t*_(24)_ = −0.215; *p* > 0.8). The distorted models were used significantly less than expected by chance with 13.66% (*t*_(24)_ = −9.995; *p* < 0.001).

#### 4.1.3 Eye movements

Table 2 shows the time slots identified by analyzing the eye movements during the filler trials. We used the last six out of ten time slots for our analysis of the eye movements during the actual problem trials. We decided to use all six slots despite the fact that two out of those did not show significant eye movements in the filler trials, because it is plausible that processing was not interrupted in between, but ran continuously after participants have understood the premise. Table 3 shows that the amount of participants showing eye movements along the given directions is not significant in neither the first nor the second premise (all *p* > 0.35), but significant during the conclusion phase (*p* < 0.05).

**Table 2:** **Analysis of eye tracking data from the first premise of all filler trials**.

Time slot	Experiment 1	Experiment 2
1	0.2353	0.6601
2	0.7297	0.8378
3	0.3143	0.4950
4	0.8286	0.0122*
5	0.0169*	0.1991
6	0.0080*	0.0082*
7	0.1388	0.0097*
8	0.0299*	0.0181*
9	0.1463	0.0000*
10	0.0404*	0.0018*

*We applied a binomial test to see whether the eye movements along the given direction (and its opposite) are above the expected value of chance (1/4) within each time slot. Significance is based on an error probability of 0.05*.

**Table 3:** **The number of participants showing significant eye movements along the given directions**.

	Experiment 1	Experiment 2
Premise 1	2 out of 25	9 out of 23*
Premise 2	2 out of 25	4 out of 23*
Conclusion	4 out of 25*	5 out of 23*

*Significance is based on an error probability of 0.05*.

**Figure 4:**
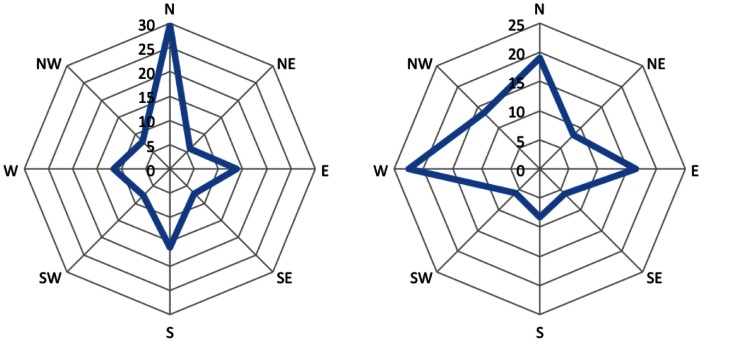
**Distribution of eye movements during first premises of the form “A is west of B.”** Amplitude represents the percentage of saccades mapped onto the respective cardinal direction.

**Figure 5:**
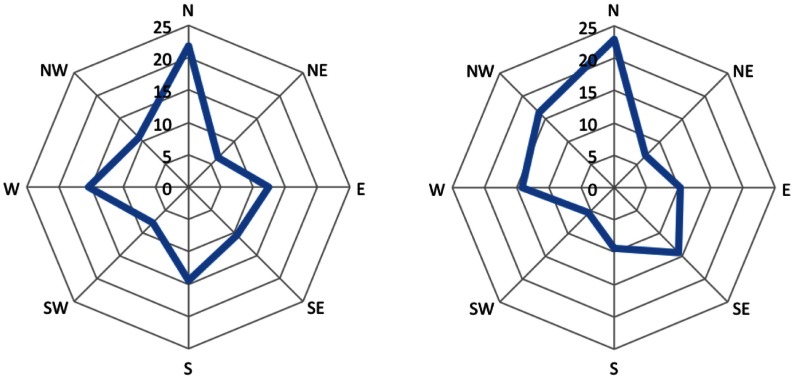
**Distribution of eye movements during first premises of the form “A is north-west of B.”** Amplitude represents the percentage of saccades mapped onto the respective cardinal direction.

The left parts of the Figures 4 and 5 show diagrams of the recorded eye movements during all first premises of the form *A is west of B* and *A is north-west of B*, respectively. It is evident that the percentage of saccades along the given direction and the opposing direction are not above chance, i.e., 12.5%, for both types of premises.

### 4.2 Experiment 2: Visual mental representation

#### 4.2.1 Participants

Thirty one undergraduate students of the University of Bremen, 15 male and 16 female, participated in the study for monetary compensation.

Eight of the 31 participants were discarded due to an error rate of more than 30% incorrectly answered trials. The remaining 23 participants comprised 12 males and 11 females.

#### 4.2.2 Preferences

**Figure 6:**
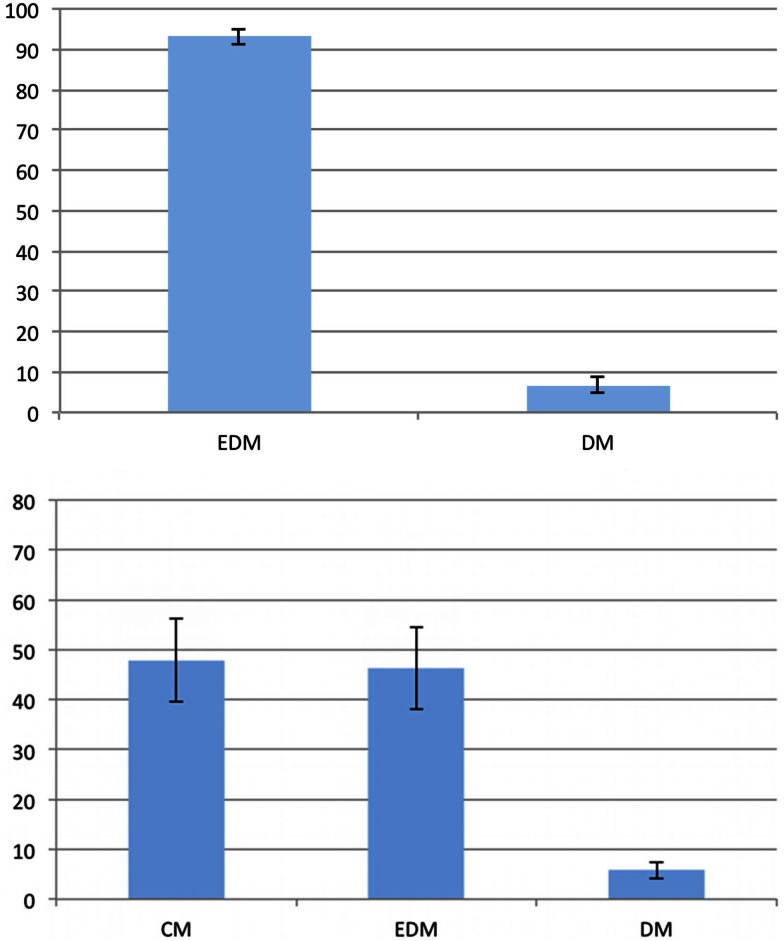
**Preferences in the second experiment**. The vertical axis represents the frequency of the given answer. Top: 90° problems; bottom: 45° problems. Error bars show the standard error of the mean. EDM, equal-distance model; CM, cardinal model; DM, distorted models.

Preferences were analyzed in the same way as in Experiment 1. We discarded those trials for which the participants gave no or an incorrect answer for the analysis of the preferences (9% of all trials). Figure 6 shows the preferences for both problem types. For the 90° problems, the equal-distance model was used in 93.2% of all trials, which shows a significant preference (*t*_(22)_ = 29.350; *p* < 0.001). Consequently, the distorted models are employed significantly below chance with 6.8% (*t*_(22)_ = −29.350; *p* < 0.001). For the 45° problems, we found a significant preference for the equal-distance model with 46.32% (*t*_(22)_ = 2.512; *p* < 0.05) as well as for the cardinal model with 47.9% (*t*_(22)_ = 2.683; *p* < 0.05). The distorted models were used significantly less compared to their expected value with 5.78% (*t*_(22)_ = −25.360; *p* < 0.001).

#### 4.2.3 Eye movements

Table 2 shows the time slots during which participants showed significant eye movements during the filler trials. Based on this, we used the last seven out of ten time slots for the eye movement analysis for the problem trials. We decided to use all seven slots despite the fact that one out of those did not contain significant eye movements, because we assumed, just as in the first experiment, that processing is not paused in between. Contrasting the first experiment, we found a significant amount of participants showing significant eye movements during all three phases (Prem. 1: *p* < 0.001; Prem. 2: *p* < 0.05; Concl.: *p* < 0.01) as shown in Table 3.

The right parts of the Figures 4 and 5 show diagrams of the recorded eye movements during all first premises of the form *A is west of B* and *A is north-west of B*, respectively. The Figures show that saccades along the given direction as well as the opposing direction are above the frequency of chance (i.e., 12.5%) for both types of premises.

#### 4.2.4 Comparison of eye-movers to non-eye-movers

Based on the literature, we expected to find spontaneous eye movements corresponding to the processed spatial relations when a visual mental representation is employed. In line with this assumption, a majority of participants exhibited systematic eye movements. We compared the participants that showed a significant amount of eye movements along the given directions in any of the phases (both premises or the conclusion) with those that did not show significant eye movements in any of the phases. Given this definition, 13 out of the 23 participants qualified as eye-movers; the 10 remaining participants will be referred to as non-eye-movers.

**Figure 7:**
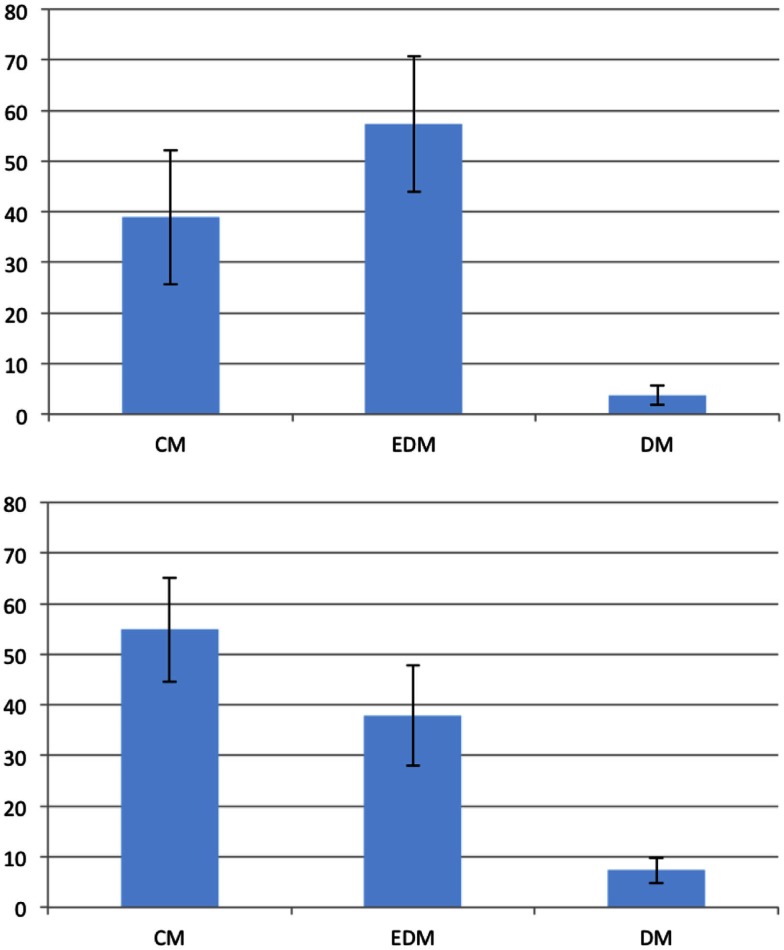
**Preferences of the 45° problems in the second experiment**. The vertical axis represents the frequency of the given answer. Top: non-eye-movers; bottom: eye-movers. Error bars show the standard error of the mean. EDM, equal-distance model; CM, cardinal model; DM, distorted models.

There was no significant difference between eye-movers and non-eye-movers regarding error rate, reaction times, and sex (all *p* > 0.19). The eye-movers and non-eye-movers, however, showed different preferences for the 45° problems as shown in Figure 7. The eye-movers showed a significant preference for the cardinal direction model with 54.84% (*t*_(12)_ = 2.7884; *p* < 0.05). The equal-distance model was not significantly preferred with 37.85% (*t*_(12)_ = 1.2527; *p* > 0.23) and the distorted models were employed significantly less than expected with 7.31% (*t*_(12)_ = −16.1961; *p* < 0.001). The non-eye-movers showed no significant preference for the cardinal direction model with 38.88% (*t*_(9)_ = 0.994; *p* > 0.34) but for the equal-distance model with 57.32% (*t*_(9)_ = 2.2926; *p* < 0.05). The distorted models were significantly below expectation with 3.8% (*t*_(9)_ = −22.3421; *p* < 0.001).

### 4.3 Comparison of the experiments

In the first experiment, it is only during the conclusion phase that the number of participants that showed systematic eye movements becomes significant. This finding seems unexpected given that the number of eye-movers of the second experiment is highest during the first premise whereas the number of eye-movers in the first experiment is not significant for neither premise. Furthermore, analysis of the eye movements should be most accurate for the first premise as participants are only aware of one spatial relation at that time and all saccades along the other directions can be assumed not to have any relation to the mental representation constructed. In contrast, during the second premise or the conclusion, all three spatial relations are (at least implicitly) available to the participant and could also result in eye movements, which would, however, not all be counted as “correct” eye movements, because we only checked for the spatial relations of the two premises during the second premise and we only checked for the relation that is given as the answer during the conclusion. Thus, the chance for finding significant eye movements during specifically the conclusion phase should be lower than for the first premise. It can, accordingly, be argued that eye movements during the conclusion phase did not necessarily result from the internal processing of spatial relations but that some participants moved their gaze in anticipation of the answer screen. The answer screen was projected on the wall just after participants clicked to indicate they found an answer. A saccade from the middle of their visual field toward the appropriate number on the answer screen, i.e., the number which represents their given answer, would have been mapped onto the cardinal direction that corresponds to their answer. Thus, there is reason to doubt that the significant number of eye-movers that we find for the conclusion phase in the first experiment is a result of the employed mental representation.

Given the lack of spontaneous eye movements along the processed relations for the non-eye-movers of the second experiment, we conclude that these participants did not employ a visual mental representation. This conclusion is based on the literature (see Section 3.2) which shows that employment of visual mental representations is related to the occurrence of such spontaneous eye movements and, furthermore, that these eye movements have a functional role in the employment of visual mental representations (Johansson et al., 2012). It may be that the non-eye-movers likely used a spatial mental representation like the participants of the first experiment; this conclusion does, however, not follow from the observation or the literature. We, therefore, remain agnostic regarding the mental representation of the non-eye-movers of the second experiment.

#### 4.3.1 Comparing reasoning with visual and spatial mental representations

As the two experiments consisted of the same task with only slightly different instructions, we compared participants across the experiments^1^. Reaction times that were outside a 2.5*SD range from the mean reaction time of the corresponding phase (first and second premise and the conclusion) were excluded from analysis (3%).

**Figure 8:**
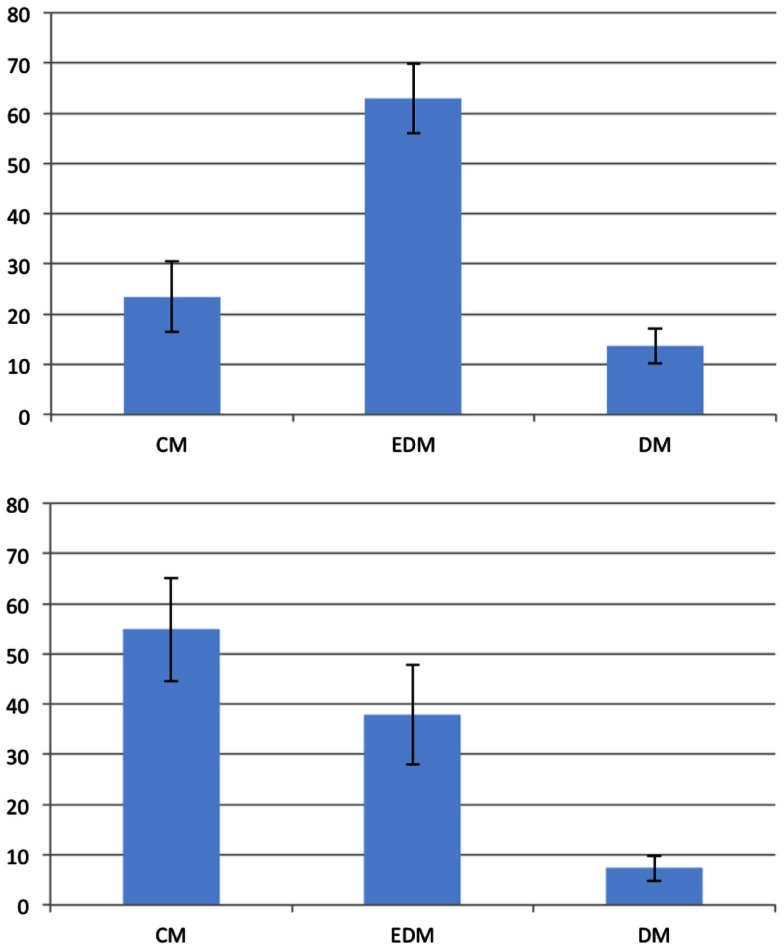
**Preferences of the 45° problems for the spatial group (top) and the visual group (bottom)**. The vertical axis represents the frequency of the given answer. Error bars show the standard error of the mean. EDM, equal-distance model; CM, cardinal model; DM, distorted models.

In order to compare the employment of visual mental representations with that of spatial mental representations, we defined two groups: the visual group and the spatial group. The spatial group comprises all participants of the first experiment. The eye-movers of the second experiment constitute the visual group. That is, the spatial group contains those participants which employed a spatial mental representation and the visual group contains those that employed a visual mental representation. There were no significant differences regarding error rate, reaction times, and sex (all *p* > 0.35) between the visual and the spatial group. However, the preferences of the two groups differed as indicated by a significant interaction between group (spatial or visual) and type of model (cardinal or equal-distance), *F*_(1,36)_ = 5.644*,p* < 0.05. Figure 8 shows the preferences of the visual and the spatial group. The spatial group showed a preference for the equal-distance model (EDM) but not for the cardinal model (CM). In contrast, the visual group showed a preference for the cardinal model (CM) but not the equal-distance model (EDM). Table 4 shows an overview of the preferences for the different groups and experiments. Interestingly, the non-eye-movers of the second experiment showed the same preferences as the participants of the first experiment, i.e., a significant preference for the equal-distance model (EDM) and no significant preference for the cardinal model (CM). This may be taken to indicate that the non-eye-movers employed a spatial mental representation despite the fact that the instructions are formulated to induce a visual mental representation.

**Table 4:** **Comparison of preferences for the 45° problems between different groups; S+, frequency significantly above chance; S−, frequency significantly below chance; NS, frequency does not significantly differ from chance; CM, cardinal model; EDM, equal-distance model; DM, distorted models**.

Group	CM	EDM	DM
Exp 1 (spatial group)	NS	S+	S−
Exp 1, eye-mover	NS	S+	S−
Exp 1, non-eye-mover	NS	S+	S−
Exp 2	S+	S+	S−
Exp 2, eye-mover (visual group)	S+	NS	S−
Exp 2, non-eye-mover	NS	S+	S−

## 5 Discussion

The conducted experiments yielded two main results. First, the employed reasoning task led to no significant systematic eye movements when a spatial mental representation was employed, i.e., for the spatial group. In contrast, we found significant systematic eye movements for a majority of the participants in the second experiment, i.e., the visual group which employed a visual mental representation. Second, there are significant preferences in the answers for the under-specified problems in both the visual and the spatial group. The preferences did, however, differ between the employed mental representations.

These results relate to the two main open issues about the relationship between spatial and visual mental representation (identified in Section 3.3): (1) whether spatial mental representations are modality-specific, and (2) whether human visuo-spatial reasoning is realized on the level of spatial mental representations.

Regarding the first issue, we observed systematic eye movements in the second experiment but not for the first experiment. The eye movements observed in second experiment, i.e., the one in which the employment of a visual mental representation was induced, corroborate several studies reporting spontaneous eye movements during visual mental imagery. The fact that we did not find these eye movements for the essentially same reasoning task in the first experiment, i.e., the one in which the employment of a spatial mental representation was induced, suggests that other (attentional) processes are employed when reasoning with spatial mental representations. Since eye movements have been found to play a functional role in processing visual mental representations (Johansson et al., 2012) and are therefore not epiphenomenal, we can conclude that reasoning with visual mental representations draws on overt attentional processes of visual perception and reasoning with spatial mental representations does not. This finding lends support to the assumption of mental model theory that spatial mental representations are amodal or multi-modal.

Regarding the second issue – whether reasoning is realized on the level of spatial mental representations – our results show different preferences depending on the employed mental representation. The visual group showed a significant preference for the cardinal model (CM) but not for the equal-distance model (EDM) for the 45° problems. In contrast, the spatial group showed a significant preference for the equal-distance model (EDM) but not for the cardinal model (CM) for the 45° problems. Mental model theory assumes that the hierarchical relationship between visual mental representations and spatial mental representations is such that reasoning happens on the level of the spatial mental representation (Knauff and Johnson-Laird, 2002; Knauff et al., 2003) specifically when visual information is irrelevant to the task at hand (as it is the case in the presented experiments). This assumption seems in contradiction to the presented results. The fact that we observed different preferences for the two mental representations for essentially the same reasoning task challenges the claim that reasoning is based on spatial mental representations. This similarly affects the theory of mental imagery which also states that visual mental representations require underlying spatial mental representations. In order to construct, inspect and reason with a visual representation, spatial information is necessary to, for example, “know” the location, size, and spatial relations of the shapes that make up a visual mental representation. The results of the experiments are thus hard to reconcile with both the mental model theory and the theory of mental imagery. The assumed hierarchical relationship between spatial and visual mental representations has to be extended with additional explanations about how spatial information is transformed or processed differently in a visual mental representation. In the following, we interpret the results on the preferences with respect to this assumption of the two theories.

The preferred answer given by participants in the spatial group was such that the spatial configuration of the problem has equal distances between the entities. In contrast, the preferred answer of the participants in the visual group was such that the spatial configuration contains distances of different length. This is especially puzzling given the assumption that those spatial relations are supposed to be provided by the spatial mental representation to the visual mental representation. Sticking with the assumption that the spatial information is provided by the underlying spatial representation, one can think of two general explanations: (1) the spatial relations are somehow altered in the context of a visual representation, or (2) the spatial relations are the same but are processed differently in the two mental representations. Regarding the first option, spatial relations might become more specified when represented in a visual mental representation (Schultheis et al., 2007). That is, additional properties such as distance are specified. On the level of the spatial mental representation, distance might only be represented with generic default values. This would fit with the preferred mental model of the spatial group, in which all distances are equal. This option is furthermore supported by an assumption of mental model theory: “[w]hen people understand spatial descriptions, they imagine symmetrical arrays in which adjacent objects have roughly equal distances between them […]” (Johnson-Laird and Byrne, 1991, p. 94). Regarding the second option, the way spatial relations are processed could differ between the two mental representations. This explanation would fit well with the fact that we found spontaneous eye movements that align with the currently processed spatial relations for the visual group, but we did not find such eye movements for the spatial group. An implementation of the second option is proposed by a new model of visuo-spatial mental imagery in which processing of spatial relations is affected by additional visual information and realized by attention shifts such as eye movements (Sima, 2011; Sima and Freksa, 2012).

## 6 Conclusion

Our experiments provided two new insights on the so far little investigated relationship between visual and spatial mental representations: (1) visual and spatial mental representations differ in their employment of overt attentional processes of visual perception, (2) there are preferences when employing visual mental representations just as for spatial mental representations, but the preferences can differ for the same reasoning task. These findings are hard to reconcile with current theories on visuo-spatial processing and challenge some of their assumptions. Future work is necessary to shed more light on the exact relationship between visual and spatial mental representations. This will have to include the refinement of the existing theoretical frameworks on the one hand as well as further empirical research on the other hand. Regarding the theories, we have additionally presented a systematic comparison of mental model theory and the theory of mental imagery. This comparison showed that the two theories that are often investigated separately likely investigate the same visual and spatial mental representations. This comparison might serve as the basis of a new unified theory combining the results achieved within both mental model theory and the theory of mental imagery. Regarding the future empirical work, the presented experiments show one way of comparing visual and spatial mental representations while keeping the experimental task essentially the same.

## Conflict of Interest Statement

The authors declare that the research was conducted in the absence of any commercial or financial relationships that could be construed as a potential conflict of interest.

## Footnotes

^1^There is evidence that the (*a priori*) differences between the two groups of the two experiments were not more substantial or qualitatively different than if the groups had resulted from random assignments within a single experiment. First, the participants of both experiments were recruited from the same population of students from the University of Bremen. The setup of the two experiments was identical apart from the variation in instructions. This includes specifically the equipment, the room, the experimenter, and the materials. The experiments were conducted within two consecutive semesters. Second, the two groups did not significantly differ with respect to sex $\left(\chi_{\left(1\right)}^{2}=1.42,p>0.2\right)$ or field of study $\left(\chi_{\left(2\right)}^{2}=1.18,p>0.5\right)$. The two groups also did not differ significantly in their age (*t*_(46)_ = –1.084; *p* > 0.28, two-tailed) or performance in the paper-folding test (*t*_(46)_ = –0.455; *p* > 0.65, two-tailed). Third, using the method described in Masson (2011), the participants age and performance in the paper-folding test, provided positive evidence for the null hypothesis that the two groups did not differ (*p*(*H*_0_|*D*) = 0.79, *p*(*H*_0_|*D*) = 0.86, respectively).
